# Data describing the accuracy of non-numerical visual features in predicting fMRI responses to numerosity

**DOI:** 10.1016/j.dib.2017.11.022

**Published:** 2017-11-09

**Authors:** Ben M. Harvey, Serge O. Dumoulin

**Affiliations:** a Faculty of Psychology and Education Sciences, University of Coimbra, Rua do Colégio Novo, 3001-802 Coimbra, Portugal; b Experimental Psychology, Helmholtz Institute, Utrecht University, Heidelberglaan 1, Utrecht 3584 CS, The Netherlands; c Spinoza Centre for Neuroimaging, Amsterdam, The Netherlands

## Abstract

Here we took several stimulus configurations that have the same numerosity progression but vary considerably in their non-numerical visual features. We collected responses to these stimuli using ultra-high-field (7T) fMRI in a posterior parietal area that responds to changes in these stimuli. We first quantify the relationships between numerosity and several non-numerical visual features in each stimulus configuration. We then use population receptive field (pRF) modeling to quantify how well responses to each of these visual features predicts the observed responses to each stimulus configuration, and observed responses to all stimulus configurations together. We compare the predictive accuracy of responses to numerosity and to non-numerical visual features in explaining the observed responses. This provides the details of the analysis outcomes summarized in an accompanying article (10.1016/j.neuroimage.2017.02.012, NIMG-16–1350).

**Specifications Table**

**Table:** 

Subject area	Biology, Psychology
More specific subject area	Cognitive neuroscience, Neuroimaging
Type of data	Figures, tables of descriptive statistics and descriptive text
How data was acquired	Ultra-high-field functional MRI, using a Philips Achieva 7T scanner
Data format	Analyzed
Experimental factors	Functional MRI scanner outputs from several scanning sessions have been motion corrected and interpolated to common anatomical models for each subject. We examine data within a particular region of interest in the posterior parietal lobe that is thought to respond to a particular stimulus feature, numerosity.
Experimental features	We use population receptive field (pRF) neural response models to capture responses to stimuli that vary in numerosity. Changes in numerosity are inevitably accompanied by changes in non-numerical visual features. We compare outcomes of numerosity response models to outcomes of alternative models of responses these non-numerical visual features.
Data source location	Utrecht, Netherlands
Data accessibility	Analyzed data is displayed within the article. Source data is too large to practically distribute (> 100 GB) and is freely available on request from the authors.

**Value of the data**

– Many studies of numerosity processing use the stimulus configurations examined here to demonstrate behavioral discriminability or neural responses to numerosity do not reflect responses to low-level visual features.
– We quantify how low-level visual features change with numerosity in these stimulus configurations.
– This allows researchers to see where behavioral and neural responses are consistent with responses to low-level visual features.
– The presented fMRI analyses distinguish in detail between recent hypotheses of responses to numerosity or co-varying non-numerical visual features.
– Data were acquired with ultra-high-field 7T fMRI, a technology unavailable to most researchers.

## 1 Data description

We took five stimulus configurations that are commonly used to investigate numerosity perception and neural responses to numerosity [1], [2], [3], [4], [5], [6], [7]. These varied in non-numerical visual features, but had identical progressions of numerosity. We took fMRI responses to these stimuli from the human posterior parietal cortex [1], [2], [4], which varied between stimulus configurations. For each stimulus configuration, we quantified how each visual feature changed with numerosity. We used the time course of visual feature progressions to predict the responses that would be expected if the fMRI recording sites were responding to these visual features, thus quantifying how well responses to each visual feature predicted the responses seen. We compared the predictive accuracy of these visual feature models with that of numerosity models. To clarify the patterns in the data that underlie the analysis outcomes, we provide textual descriptions for each visual feature to show how these patterns led to the observed outcomes.

**Table 1:** Differences between stimulus features in mean, standard deviation, and correlation with numerosity.

Feature and stimulus configuration	Mean	Standard deviation	Standard deviation/mean	Correlation with log(numerosity) (*R*)
**Feature: log(Numerosity)**				
Any configuration	0.761	0.445	0.585	1
**Feature: Individual item area**				
Constant area	0.018 deg^2^	0.019 deg^2^	1.064	−0.890
Constant item size	0.021 deg^2^	0 deg^2^	0	0
Constant perimeter	0.200 deg^2^	0.405 deg^2^	2.023	−0.741
High density	0.018 deg^2^	0.019 deg^2^	1.064	−0.890
Variable features	0.013 deg^2^	0 deg^2^	0	0
All configurations	0.054 deg^2^	0.189 deg^2^	3.503	−0.338
All except constant	0.078 deg^2^	0.242 deg^2^	2.693	−0.442
**Feature: Individual item perimeter**				
Constant area	0.419 deg	0.220 deg	0.525	−0.969
Constant item size	0.516 deg	0 deg	0	0
Constant perimeter	1.115 deg	1.187 deg	1.064	−0.890
High density	0.419 deg	0.220 deg	0.525	−0.969
Variable features	0.410 deg	0 deg	0	0
All configurations	0.576 deg	0.594 deg	1.030	−0.479
All except constant	0.651 deg	0.761 deg	1.168	−0.627
**Feature: Total item area**				
Constant area	0.064 deg^2^	0 deg^2^	0	0
Constant item size	0.187 deg^2^	0.168 deg^2^	0.901	0.934
Constant perimeter	0.361 deg^2^	0.384 deg^2^	1.064	−0.890
High density	0.064 deg^2^	0 deg^2^	0	0
Variable features	0.118 deg^2^	0.106 deg^2^	0.901	0.934
All configurations	0.159 deg^2^	0.217 deg^2^	1.366	−0.076
All except constant	0.222 deg^2^	0.263 deg^2^	1.183	−0.105
**Feature: log(Total item perimeter)**				
Constant area	0.332 deg	0.223 deg	0.671	1
Constant item size	0.474 deg	0.445 deg	0.940	1
Constant perimeter	0.609 deg	0 deg	0	0
High density	0.332 deg	0.223 deg	0.671	1
Variable features	0.485 deg	0.444 deg	0.916	1
All configurations	0.446 deg	0.320 deg	0.716	0.801
All except constant	0.406 deg	0.346 deg	0.853	0.926

**Table 2:** Differences between stimulus features in mean, standard deviation, and correlation with numerosity.

Feature and stimulus configuration	Mean	Standard deviation	Standard deviation/mean	Correlation with log(numerosity) (*R*)
**Feature: log(Numerosity)**				
Any configuration	0.761	0.445	0.585	1
**Feature: log(Convex hull area)**				
Constant area	0.511 deg	0.204 deg	0.400	0.775
Constant item size	0.493 deg	0.282 deg	0.573	0.756
Constant perimeter	0.598 deg	0.028 deg	0.046	0.671
High density	0.257 deg	0.115 deg	0.448	0.842
Variable features	0.493 deg	0.282 deg	0.573	0.756
All configurations	0.470 deg	0.229 deg	0.488	0.585
**Feature: log(Convex hull perimeter)**				
Constant area	−0.176 deg^2^	0.419 deg^2^	−2.383	0.865
Constant item size	−0.211 deg^2^	0.576 deg^2^	−2.731	0.839
Constant perimeter	0.047 deg^2^	0.076 deg^2^	1.598	0.532
High density	−0.647 deg^2^	0.235 deg^2^	−0.363	0.922
Variable features	−0.211 deg^2^	0.576 deg^2^	−2.731	0.839
All configurations	−0.239 deg^2^	0.466 deg^2^	−1.946	0.652

**Table 3:** Differences between stimulus features in mean, standard deviation, and correlation with numerosity.

Feature and stimulus configuration	Mean	Standard deviation	Standard deviation/mean	Correlation with log(numerosity) (*R*)
**Feature: log(Numerosity)**				
Any configurations	0.761	0.445	0.585	1
**Feature: Luminance density**				
Constant area	0.178	0.296	1.660	−0.709
Constant item size	0.267	0.271	1.016	−0.391
Constant perimeter	0.339	0.330	0.973	−0.936
High density	0.334	0.251	0.753	−0.816
Variable features	0.167	0.168	1.005	−0.388
All configurations	0.257	0.268	1.043	−0.640
**Feature: log(Edge density)**				
Constant area	0.508 deg^−1^	0.252 deg^−1^	0.470	−0.554
Constant item size	0.685 deg^−1^	0.316 deg^−^^1^	0.461	−0.121
Constant perimeter	0.562 deg^−1^	0.076 deg^−^^1^	0.135	−0.532
High density	0.979 deg^−1^	0.091 deg^−1^	0.093	0.064
Variable features	0.696 deg^−1^	0.317 deg^−1^	0.455	−0.122
All configurations	0.686 deg^−1^	0.280 deg^−1^	0.408	−0.172
**Feature: Numerical density**				
Constant area	9.789 deg^−^^2^	4.952 deg^−2^	0.506	0.375
Constant item size	12.560 deg^−2^	12.756 deg^−2^	1.016	−0.391
Constant perimeter	7.264 deg^−2^	5.708 deg^−2^	0.786	0.951
High density	29.951 deg^−2^	18.499 deg^−^^2^	0.618	0.899
Variable features	12.560 deg^−2^	12.756 deg^−2^	1.016	−0.391
All configurations	14.425 deg^−2^	14.092 deg^−2^	0.977	0.189

**Table 4:** Differences between stimulus features in mean, standard deviation, and correlation with numerosity.

Feature and stimulus configuration	Mean	Standard deviation	Standard deviation/mean	Correlation with log (numerosity) (*R*)
**Feature: log(Numerosity)**				
Any configuration	0.761	0.445	0.585	1
**Feature: Display RMS contrast**				
Constant area	0.186	0	0	0
Constant item size	0.272	0.118	0.434	0.987
Constant perimeter	0.330	0.110	0.336	−0.954
High density	0.186	0	0	0
Variable features	0.222	0.102	0.458	0.983
All configurations	0.239	0.099	0.413	0.216
All except constant	0.275	0.115	0.419	0.311
**Feature: Convex hull RMS contrast**				
Constant area	0.239	0.107	0.447	0.069
Constant item size	0.336	0.135	0.402	0.794
Constant perimeter	0.323	0.155	0.480	−0.058
High density	0.384	0.140	0.365	0.365
Variable features	0.325	0.077	0.238	0.089
All configurations	0.321	0.130	0.403	0.242

**Table 5:** Differences between stimulus features in mean, standard deviation, and correlation with numerosity.

Feature and stimulus configuration	Mean	Standard deviation	Standard deviation/mean	Correlation with log(numerosity) (*R*)
**Feature: log(Numerosity)**				
Any configuration	0.761	0.445	0.585	1
**Feature: log(High frequency contrast energy)**				
Constant area	1.472	0.024	0.016	-0.560
Constant item size	1.514	0.056	0.038	0.989
Constant perimeter	1.560	0.075	0.048	-0.991
High density	1.520	0.012	0.008	0.561
Variable features	1.492	0.060	0.040	0.993
All configurations	1.512	0.057	0.038	0.118

## 2 Experimental design, materials and methods

A complete description of our methods is included in our recent article [2]. Here we summarize important details for interpreting the presented data. We collected ultra-high-field (7T) fMRI data from eight normal subjects while presenting several stimulus configurations that each varied in numerosity identically over time. Experiments were undertaken with the informed written consent of each subject. All experimental procedures were cleared by the ethics committee of University Medical Center Utrecht. Different stimulus configurations (Fig. 1 of [2]) were designed to keep specific visual features (individual item size, total item area, or total item perimeter) constant across all numerosities, to group items more densely, or to use various shapes as items. For every stimulus configuration and numerosity, we quantified several visual features of the presented stimuli. This revealed how each visual feature changed over time in each stimulus configuration. The resulting time course of each visual feature allowed us to fit population receptive field (pRF) neural response models for each visual feature at each fMRI recording site (voxel) in a right posterior parietal area that responds to changes in these stimuli. For each visual feature, a forward model predicted neural responses at each stimulus time point depending on the displayed visual feature quantity. The neural response model described a Gaussian tuned response to the candidate visual feature, characterized by a preferred feature quantity (mean of the Gaussian distribution) and tuning width (standard deviation of the Gaussian). The overlap of the displayed visual feature quantity at each time point with this neural response model predicts the neuronal response time course. Convolving this with a hemodynamic response function (HRF), predicts the fMRI time course. FMRI time courses were predicted for a large range of preferred visual feature quantity and tuning width parameter combinations. For each recording site and each visual feature, the parameters were chosen from the prediction that best fit the data by minimizing the sum of squared errors (and so maximizing *R*^2^) between the predicted and observed fMRI time courses.

This R^2^ quantified the amount of variance in each recording site's response that was explained by each neural response model for each visual feature (‘variance explained’). We determined the distribution of variance explained by every neural response model in every stimulus configuration (configuration-specific models). As neural response functions are unlikely to change between stimulus configurations, we also constrained neural response models to use the same preferred visual feature quantity and tuning width parameters to predict responses to all stimulus configurations (constrained models). To determine whether neural tuning for a particular visual feature explained responses better than tuning for numerosity, we performed Wilcoxon signed rank tests, comparing the variance explained by the numerosity response models in each recording site to that explained by visual feature response models at the same site.

## 3 Data

### 3.1 Individual item area, individual item perimeter, total item area and total item perimeter

Responses to these features made identical predictions to responses to individual item luminance, individual item radius, and total item luminance and total item radius respectively. As the high density configuration used the same item sizes as the constant area configuration, some visual features had identical magnitudes in both these configurations. Similarly, items had a constant size across numerosities in the constant size and variable features configurations, so some visual features had very similar magnitudes in both these configurations.

**Fig. 1:**
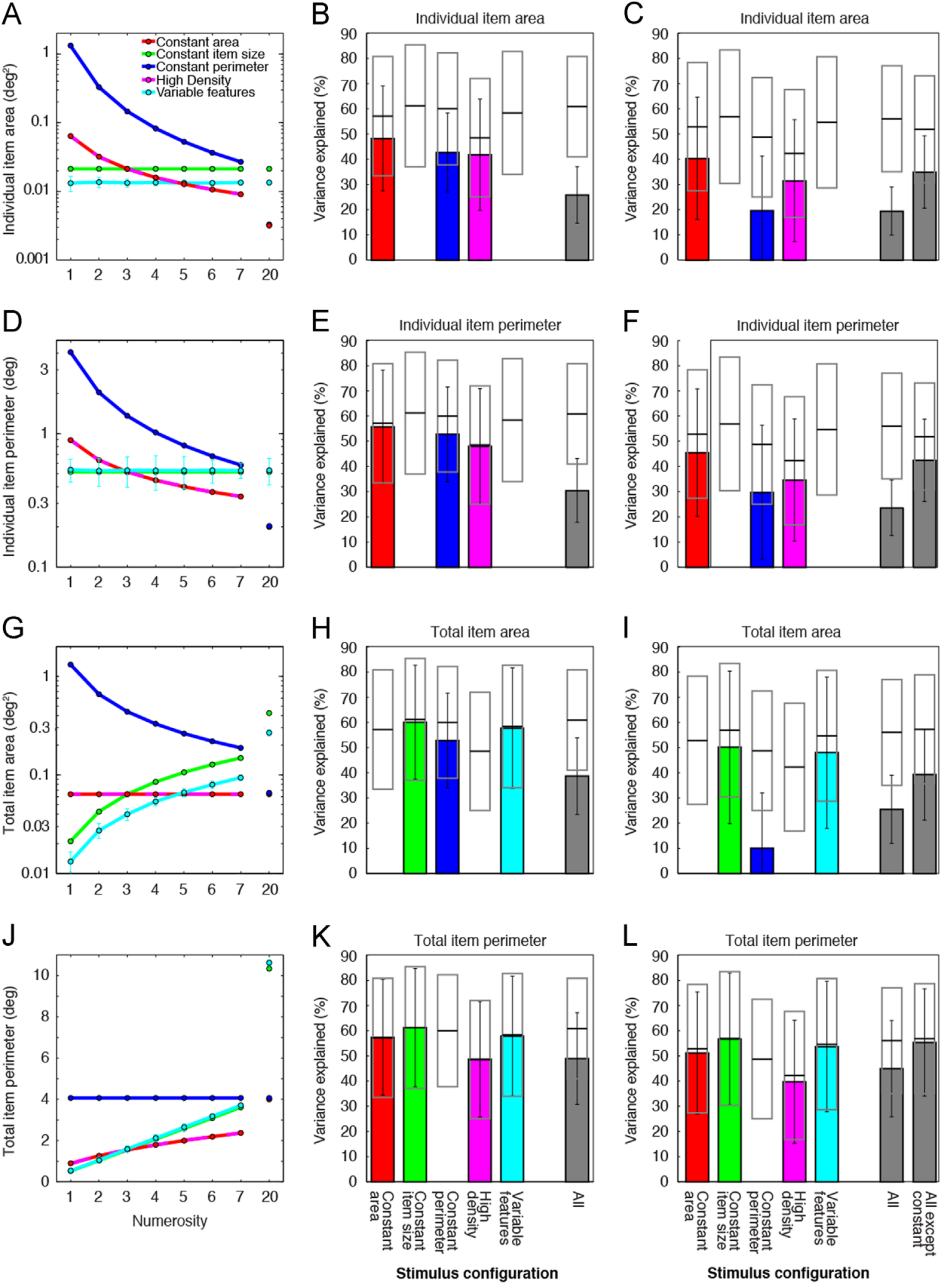
Models of responses to individual item area, individual item perimeter, total item area and total item perimeter predicted less response variance than models of responses to numerosity did. (A, D, G and J) In some stimulus configurations, areas and perimeters co-varied approximately linearly with numerosity. In others, they varied nonlinearly with numerosity or did not vary. As such, numerosity models and area or perimeter models made different predictions that explained response variance to different extents. Colored points and lines show the areas and perimeters for each numerosity in each stimulus configuration. Where areas and perimeters had different values on different presentations, colored points show the mean magnitude and error bars show the standard deviation. (B, E, H and K) In stimulus configurations where areas and perimeters co-varied approximately linearly with numerosity, area and perimeter models predicted responses approximately as well as numerosity models did. Where they varied nonlinearly with numerosity, area and perimeter models consistently predicted less responses variance than numerosity models did. Where areas and perimeters were constant for all numerosities within a stimulus configuration, they predicted no response variance. Over all stimulus configurations, numerosity models predicted responses far better than area or perimeter models did. (C, F, I and L) When all response models were constrained to use identical parameters to predict responses to all stimulus configurations, area and perimeter models predicted less response variance than separate models fit to individual stimulus configurations (shown in B, E, H and K) did, particularly when visual features in different stimulus configurations co-varied with numerosity over different ranges or in different directions. Bars show the mean variance explained in responses at many recording sites, and error bars show the standard deviation. Black lines show the mean response variance explained by numerosity models in each stimulus configuration, and gray boxes represent the standard deviation, taken from Fig. 2 of [2].

Individual item area and perimeter models explained responses well only for the constant area, constant perimeter and high density stimulus configurations, where item size co-varied with numerosity (Fig. 1A–F, Table 1). Nonlinear changes in individual item area and perimeter within the constant perimeter configuration (Fig. 1A and D) did not predict responses as well as numerosity (Fig. 1B and E). Furthermore, different ranges of individual item area and perimeter in different stimulus configurations did not predict responses well in constrained models. Explained variance fell particularly in the constant perimeter stimulus configuration (Fig. 1C and F), which covered a different range from the other configurations (Fig. 1A and D). As individual item area and perimeter did not vary in the constant item size and variable features stimulus configurations, these visual feature models explained no variance here. Whether using configuration-specific (Fig. 1B and E) or constrained (Fig. 1C and F) models, responses to individual item area or perimeter explained far less response variance than numerosity models did, when considered across all stimulus configurations (Wilcoxon signed-rank tests: *p*<10^–16^ in all cases). If only considering stimulus configurations where individual item area and perimeter varied with numerosity, responses to individual item area or perimeter still explained far less response variance than numerosity models did (*p*<10^–11^ in both cases).

Similarly, total item area models explained responses to the constant item size and variable features stimulus configurations well (Fig. 1H). Total item area co-varied approximately linearly with numerosity in both configurations (Fig. 1G), so changes in total item area followed changes in numerosity here. Total item area varied nonlinearly with numerosity in the constant perimeter stimulus configuration (Fig. 1G), predicting responses less well than numerosity. Constrained response models explained little variance in the constant perimeter configuration (Fig. 1I) because total item area and numerosity correlated negatively in this stimulus configuration, but positively in the constant item size and variable features configurations (Table 1). As total item area did not vary in the constant area and high density stimulus configurations, total item area models explained no variance here. Whether using configuration-specific (Fig. 1H) or constrained (Fig. 1I) response models, the total item area model explained far less response variance than the numerosity model did, when considered across all stimulus configurations (*p*<10^–16^ in both cases). If only considering stimulus configurations where total item area varied with numerosity, total item area models still explained far less response variance than numerosity models did (*p*<10^–16^).

As total item perimeter co-varied approximately linearly with numerosity in all except the constant perimeter condition (Fig. 1J), configuration-specific total item perimeter models gave similar results to numerosity models in all of these conditions (Fig. 1K). However, the relationship between numerosity and total item perimeter differed between these conditions, and constrained total item perimeter models predicted responses less well than constrained numerosity models did (Fig. 1L). Critically, as total item perimeter did not vary in the constant perimeter stimulus configuration, these models explained no variance here. Responses to the constant perimeter configuration varied similarly to responses to other stimulus configurations. Whether using configuration-specific (Fig. 1K) or constrained (Fig. 1L) models, total item perimeter models explained far less response variance than numerosity models did, when considered across all stimulus configurations (*p*<10^–20^ in both cases). Even if all responses to the constant perimeter configuration were excluded from these analyses, constrained total item perimeter models still explained significantly less response variance than constrained numerosity models did (*p*<10^-6^).

### 3.2 Convex hull perimeter and convex hull area

The convex hull is the smallest convex line that surrounds all items of the set. This can be visualized as a rubber band stretched around the set. We quantified the length of this line (convex hull perimeter), and the area within it (convex hull area). Both had similar relationships to numerosity, but neither was geometrically related to numerosity as both depend on random item placement.

**Fig. 2:**
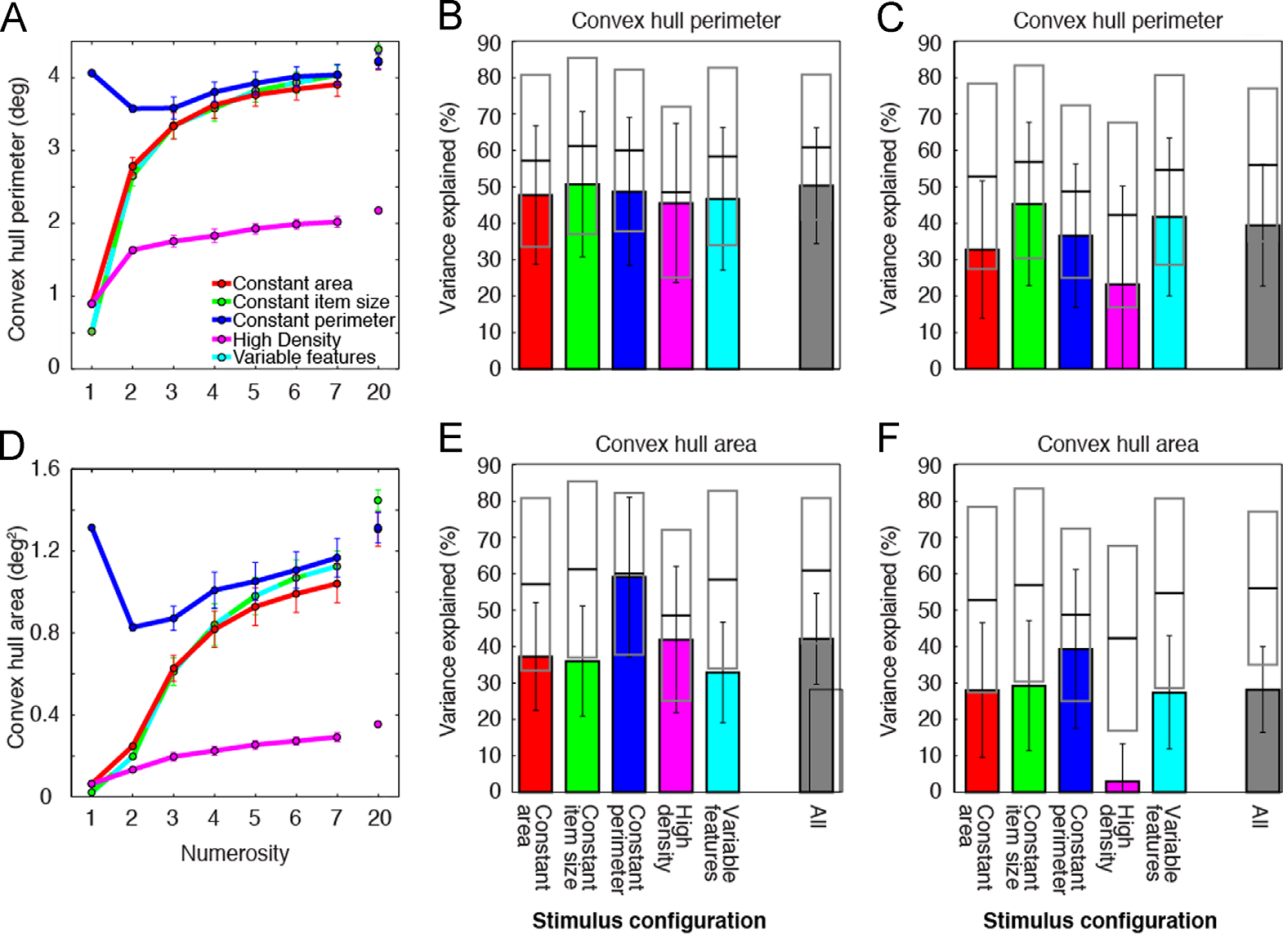
Models of responses to convex hull perimeter and convex hull area predicted less response variance than models of responses to numerosity did. (A and D) Convex hull extent varied nonlinearly with numerosity in all stimulus configurations. (B and E) Convex hull extent models consistently predicted less response variance than numerosity models did, due to these nonlinear relationships. Over all stimulus configurations, numerosity models predicted responses far better than convex hull perimeter or area models did. (C and F) When all response models were constrained to use identical parameters to predict responses to all stimulus configurations, convex hull extent models predicted less response variance than separate models fit to individual stimulus configurations (shown in B and E) did, particularly for the high density stimulus configuration, where convex hull extents had very different ranges to the other stimulus configurations (Table 2).

Both convex hull perimeter and area typically increased with increasing numerosity, but followed complex nonlinear relationships in all stimulus configurations (Fig. 2A and D, Table 2). Responses to these visual features predicted some response variance in every stimulus configuration, but always less than numerosity response models did (Fig. 2B and E). Convex hull perimeter and area were far lower in the high-density configuration than in other configurations (Fig. 2A and D, Table 2), and constrained models predicted these responses particularly poorly (Fig. 2C and F). Whether using configuration-specific (Fig. 2B and E) or constrained (Fig. 2C and F) models, convex hull perimeter and area models explained far less response variance than numerosity models did, when considered across all stimulus configurations (*p*<10^–16^ in all cases).

### 3.3 Luminance density, edge density and numerical density

Because the total possible stimulus area was the same for all stimuli, luminance density, edge density and numerical density within the total stimulus area were proportional to total item area, total perimeter, and numerosity respectively. This yielded the same predictive accuracy in general linear models. Therefore, we quantified luminance density, edge density and numerical density within the convex hull.

**Fig. 3:**
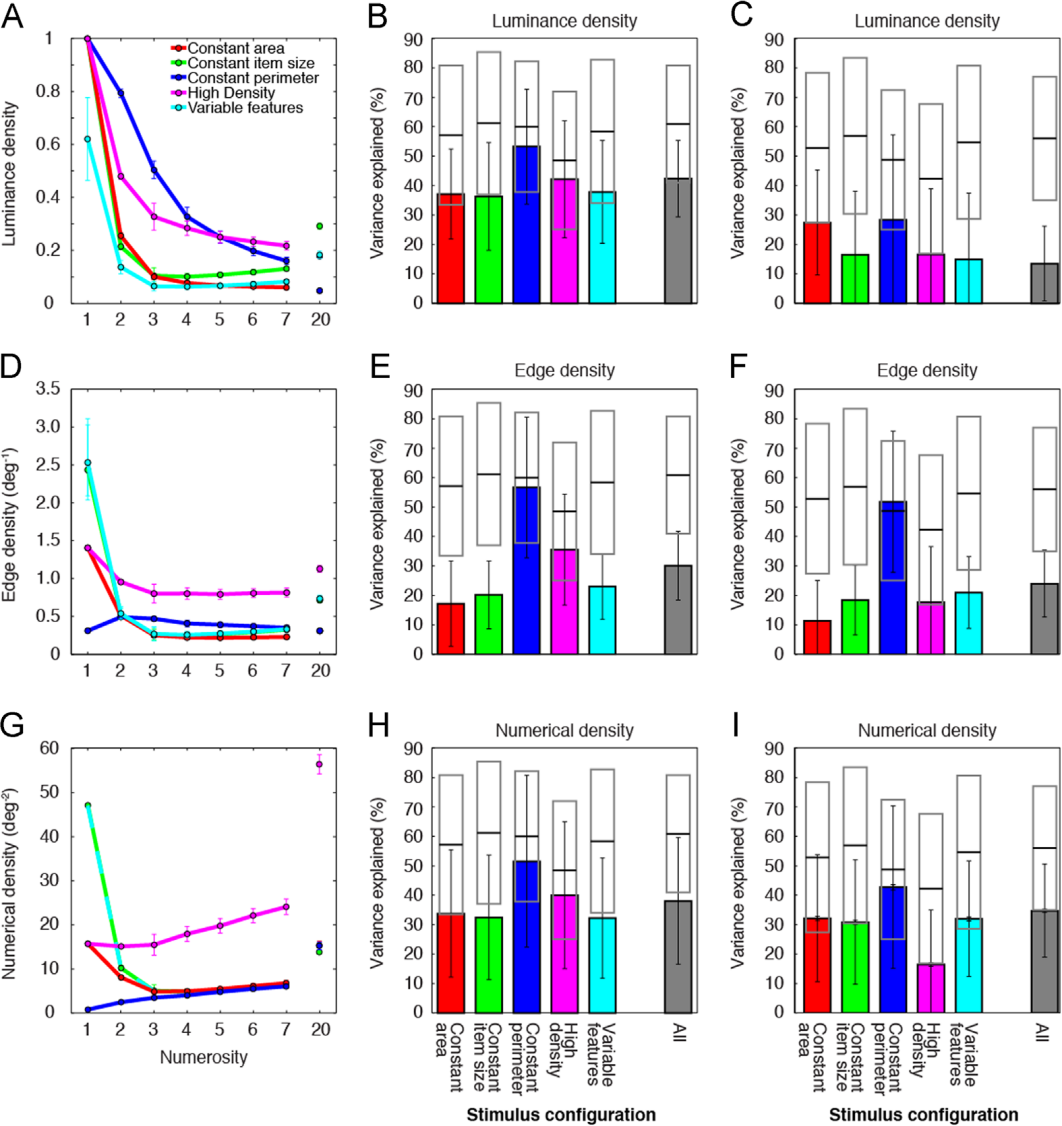
Models of responses to luminance density, edge density and numerical density predicted less response variance than models of responses to numerosity did. (A, D and G) Densities varied nonlinearly with numerosity in all stimulus configurations. (B, E and H) Density models consistently explained less response variance than numerosity models, due to these nonlinear relationships. Over all stimulus configurations, numerosity models predicted responses far better than density models did. (C, F and I) When all response models were constrained to use identical parameters to predict responses to all stimulus configurations, these models predicted less response variance than separate models fit to individual stimulus configurations (shown in B, E and H) did.

Luminance density decreased monotonically but nonlinearly with increasing numerosity in the constant perimeter and high density stimulus configuration (Fig. 3A, Table 3). This nonlinearity predicted less response variance than numerosity models did (Fig. 3B). For the other stimulus configurations, luminance density decreased rapidly with increasing numerosities up to three, then either decreased much more slowly, or increased, with increasing numerosity (Fig. 3A). The luminance densities of these stimulus configurations predicted far less response variance in single stimulus configurations than their numerosities did (Fig. 3B). Furthermore, luminance densities covered very different ranges between stimulus configurations (Table 3), and constrained models fit to all stimulus configurations captured response variance poorly because responses did not follow the different resulting predictions (Fig. 3C). Whether using configuration-specific (Fig. 3B) or constrained (Fig. 3C) response models, luminance density models explained far less response variance than numerosity models did, when considered across all stimulus configurations (*p*<10^–16^ in both cases).

Except in the constant perimeter configuration, edge density and numerical density decreased rapidly with increasing numerosities up to three, then either changed little, or increased slightly with increasing numerosity (Fig. 3D and G). These non-linear progressions predicted little of the response variance in single stimulus configurations (Fig. 3E and H). In the constant perimeter configuration, edge density and numerical density co-varied approximately linearly with numerosity over most (or all) of the numerosity range (Fig. 3D and G). Therefore, edge density and numerical density models predicted response variance well for this stimulus configuration, though less well than numerosity models did (Fig. 3E and H). Both edge density and numerical density differed widely between stimulus configurations, and constrained models incorporating these differences predicted responses poorly (Fig. 3F and I). Uniquely, constrained edge density models predicted responses to the constant perimeter configuration better than constrained numerosity models did. Numerosity model parameters changed to capture variance in responses to other stimulus configurations, while edge density models could capture little variance in other configurations so optimized their parameters for the constant perimeter configuration. Despite good prediction of responses to this single stimulus configuration, both edge density and numerical density models explained far less response variance than numerosity models did, when considered across all stimulus configurations, whether using configuration-specific (Fig. 3E and H) or constrained (Fig. 3F and I) models (*p*<10^–16^ in all cases).

### 3.4 Display RMS contrast and convex hull RMS contrast

Display RMS contrast summarizes the distribution of luminance intensities in the entire display area. Convex hull RMS contrast summarizes this distribution within the convex hull, which depended on the random placement of the items and reflected changes in convex hull area with numerosity.

**Fig. 4:**
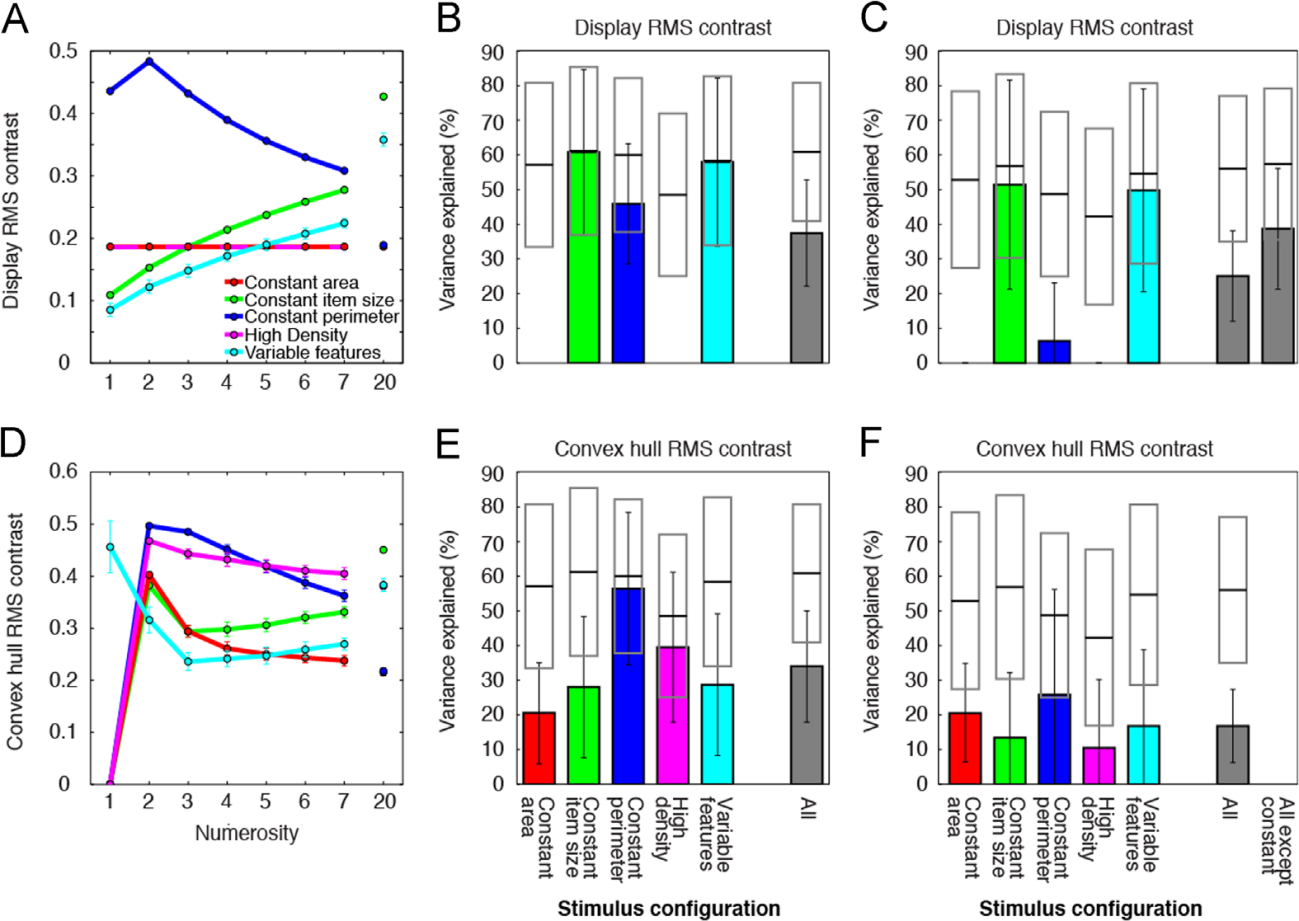
Models of responses to display RMS contrast and convex hull RMS contrast predicted less response variance than models of responses to numerosity. (A, B and C) As Fig. 3 of [2]. (D) Convex hull RMS contrast varied nonlinearly with numerosity in all stimulus configurations. (E) Convex hull RMS contrast models consistently explained less response variance than numerosity models did, due to these nonlinear relationships. Over all stimulus configurations, numerosity models predicted responses far better than convex hull RMS contrast models did. (F) When all response models were constrained to use identical parameters to predict responses to all stimulus configurations, these models predicted far less response variance than separate models fit to individual stimulus configurations (shown in E), because convex hull RMS contrast co-varied with numerosity over different ranges in different stimulus configurations (Table 4).

Much like total item area, display RMS contrast models only explained responses to the constant item size, constant perimeter and variable features stimulus configurations, where display RMS contrast co-varied approximately linearly with numerosity (Fig. 4A and B, Table 4). However, constrained response models explained little variance in the constant perimeter configuration because display RMS contrast and numerosity correlated negatively in this stimulus configuration, but positively in the constant item size and variable features stimulus configurations (Fig. 4C, Table 4). As display RMS contrast did not vary in the constant area and high density stimulus configurations, these models explained no variance here. Whether using configuration-specific (Fig. 4B) or constrained (Fig. 4C) models, display RMS contrast models explained far less response variance than numerosity models did, when considered across all stimulus configurations (*p*<10^–16^ in both cases). If only considering stimulus configurations where display RMS contrast varied with numerosity, display RMS contrast models still explained far less response variance than numerosity models did (*p*<10^–16^).

Convex hull RMS contrast followed a complex pattern of increases and decreases with changing numerosity in all stimulus configurations (Fig. 4D). Convex hull RMS contrast models predicted responses to all stimulus configurations poorly, though better for the constant perimeter and high density configurations (Fig. 4E), where convex hull RMS contrast decreased approximately linearly with increasing numerosities above two (Fig. 4D). The range of convex hull RMS contrast differed considerably between stimulus configurations (Table 4), and these differences did not predict responses well in constrained models (Fig. 4F). Whether using configuration-specific (Fig. 4E) or constrained (Fig. 4F) models, convex hull RMS contrast models explained far less response variance than numerosity models did, when considered across all stimulus configurations (*p*<10^–16^ in both cases).

### 3.5 High spatial frequency contrast energy

Spatial frequency distributions can be described by many variables: we did not test all possible spatial frequency analyses here. We tested a specific analysis proposed by previous studies [8], [9]. This used the contrast energy at high spatial frequencies to perform numerosity discriminations. In those experiments, all items had the same size, like our constant item size configuration. The range of spatial frequencies involved depended on display parameters, and we used all spatial frequencies above 4 cycles/degree.

**Fig. 5:**
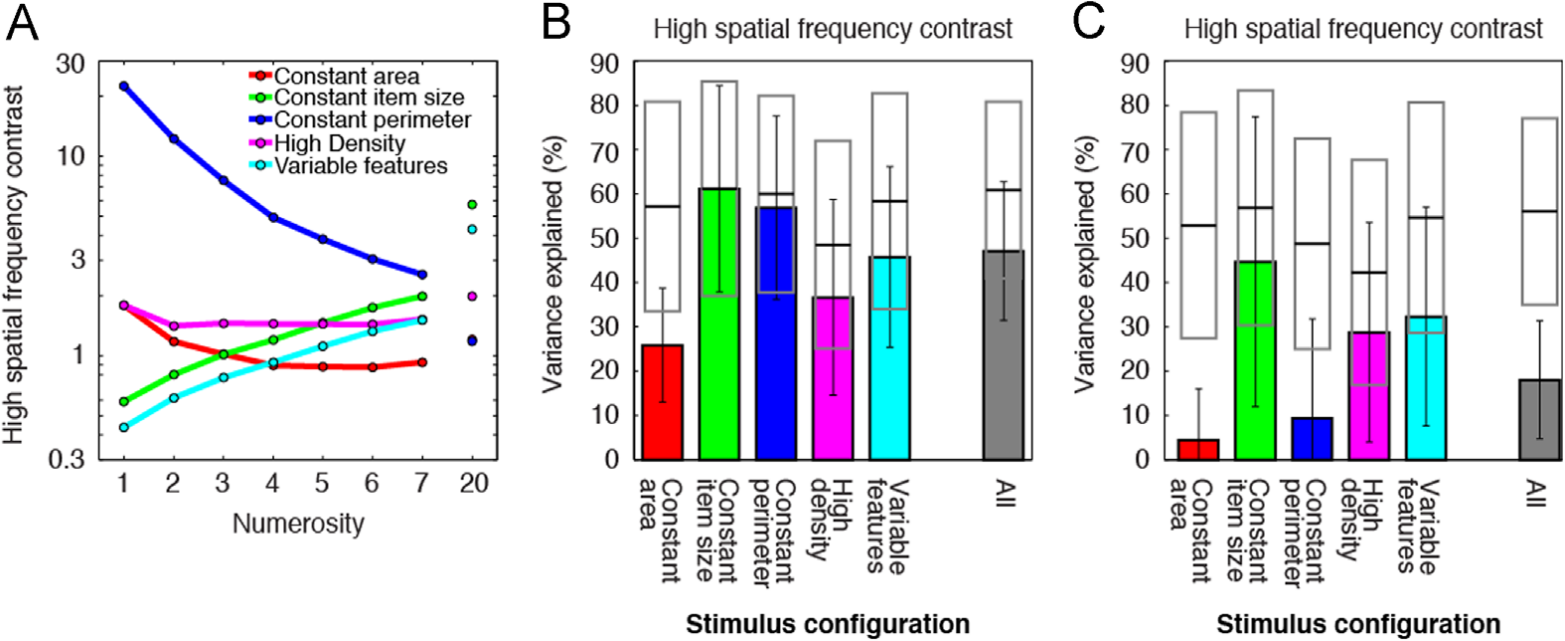
Models of responses to high spatial frequency contrast energy predicted less response variance than models of responses to numerosity did. (A) High spatial frequency contrast energy increased approximately linearly with numerosity [8], [9] when items did not change size with numerosity, in the constant item size and variable features stimulus configurations. However, high spatial frequency contrast energy decreased with increasing numerosity in the constant perimeter stimulus configuration, and first decreased slightly then increased slightly with increasing numerosity in the remaining stimulus configurations. (B) In stimulus configurations where high spatial frequency contrast energy increased or decreased approximately linearly with numerosity, high spatial frequency contrast energy models predicted responses approximately as well as numerosity models did. Where high spatial frequency contrast energy varied nonlinearly with numerosity, high spatial frequency contrast energy models consistently predicted less response variance than numerosity models did. Over all stimulus configurations, numerosity models predicted responses far better than high spatial frequency contrast energy models. (C) When all response models were constrained to use identical parameters to predict responses to all stimulus configurations, these models predicted far less response variance than separate models fit to individual stimulus configurations (shown in B), because high spatial frequency contrast energy increased with numerosity in some stimulus configurations, and decreased with numerosity in others (Table 5).

High spatial frequency contrast energy increased approximately linearly with numerosity in the constant item size stimulus configuration (Fig. 5A, Table 5), confirming previous results [8], [9]. High spatial frequency contrast energy models predicted responses to this stimulus configuration as well as numerosity models did (Fig. 5B). As items also kept the same size for all numerosities in the variable features configuration, high spatial frequency contrast energy again increased approximately linearly with numerosity here (Fig. 5A). Again, high spatial frequency contrast energy models predicted responses well (Fig. 5B).

High spatial frequency contrast energy decreased with increasing numerosity in the constant perimeter configuration (Fig. 5A). Models fit to this stimulus configuration alone also predicted responses well (Fig. 5B). However, in the constant area and high-density stimulus configurations, high spatial frequency contrast energy increased and decreased across the numerosity range (Fig. 5A). These changes predicted responses to these stimulus configurations poorly (Fig. 5B).

High spatial frequency contrast energy increased with increasing numerosity in some stimulus configurations, decreased in others, and had a large range between stimulus configurations (Table 5). These differences are not reflected in responses to these stimulus configurations: constrained models predicted responses to all stimulus configurations poorly (Fig. 5F). Indeed, high spatial frequency contrast energy only predicted responses slightly better than total item area or display RMS contrast, which had similar relationships to numerosity. Whether using configuration-specific (Fig. 5B) or constrained (Fig. 5C) response models, high spatial frequency contrast energy models explained far less response variance than numerosity models did, when considered across all stimulus configurations (*p*<10^–16^ in both cases).
